# Examining Mediators of the Relationship Between Community Mobilization and HIV Incidence Among Young South African Women Participating in the HPTN 068 Study Cohort

**DOI:** 10.1007/s10461-021-03491-7

**Published:** 2021-10-19

**Authors:** Anna M. Leddy, Torsten B. Neilands, Rhian Twine, Kathleen Kahn, Jennifer Ahern, Audrey Pettifor, Sheri A. Lippman

**Affiliations:** 1 Division of Prevention Science, Department of Medicine, Center for AIDS Prevention Studies, University of California, San Francisco, 550 16th St., 3rd Floor, San Francisco, CA 94158 USA; 2 MRC/Wits Rural Public Health and Health Transitions Research Unit (Agincourt), School of Public Health, Faculty of Health Sciences, University of the Witwatersrand, Johannesburg, South Africa; 3 Umeå Centre for Global Health Research, Division of Epidemiology and Global Health, Department of Public Health and Clinical Medicine, Umeå University, Umeå, Sweden; 4 Division of Epidemiology and Biostatistics, School of Public Health, University of California Berkeley, Berkeley, CA USA; 5 Department of Epidemiology, Gillings School of Global Public Health, University of North Carolina at Chapel Hill, Chapel Hill, NC USA

**Keywords:** Community mobilization, Adolescent girls and young women, Hope for the future, HIV prevention, South Africa

## Abstract

We previously demonstrated that village community mobilization (CM) was associated with reduced HIV incidence among adolescent girls and young women (AGYW) in South Africa. Little remains known about the mechanisms linking CM to HIV incidence. Using longitudinal data from 2292 AGYW in the HPTN 068 cohort (2011–2017), we examined whether school attendance, pro-social engagement, and hope for the future mediated the relationship between CM and HIV incidence. CM was measured at the village-level via two population-based surveys (2012 and 2014). Mediators and incident HIV infection were measured through HPTN 068 surveys and HIV testing. Mediation analyses were conducted using M*plus* 8.5, adjusting for village-level clustering and covariates. Hope for the future mediated the relationship between CM and HIV incidence (indirect effect-RR 0.98, bias-corrected 95% CI 0.96, 0.99). Pro-social engagement and school attendance did not demonstrate indirect effects. CM reduces AGYW’s HIV acquisition risk, in part, by engendering hope.

## Introduction

Adolescent girls and young women (AGYW) ages 15 to 24 years in sub-Saharan Africa account for 25% of all new HIV infections globally [1]. The highest HIV incidence rates among AGYW occur in South Africa [2], indicating the urgent need to prevent new infections among this population. Central to HIV prevention efforts must be an emphasis on addressing the social environment (i.e. the socio-cultural context in which people interact), which plays a critical role in shaping HIV risk behaviors [3]. For example, social cohesion (e.g. solidarity among a group/community [4]) and social capital (e.g. trust, norms, social control, and mutual assistance available to members of a community [5, 6]) have been associated with lower rates of early sexual debut and increased condom use [7–9]. The transition from adolescence to adulthood has been identified as a period of time when the social environment may play a particularly prominent role in determining HIV risk compared to other stages of life [10–12]. During adolescence, increasing social connection to the community and engagement in prosocial activities, such as school and sports groups, have been associated with reduced HIV risk behaviors including condom use, the number of sexual partners, early sexual debut and substance use [13–15].

Community mobilization (CM) a process whereby community members take collective action to achieve a common goal-has emerged as a promising strategy to address aspects of the social environment that contribute to HIV risk. Our group previously developed a conceptual model and measure of CM to facilitate community engagement and identify aspects of the social environment that influence HIV outcomes [16, 17]. Defining CM as comprising seven domains (shared concerns, critical consciousness, organizational structures/links, leadership, collective action, social cohesion and social control) [16], we documented some of the first evidence of its association with reduced HIV incidence among AGYW in South Africa (Box 1) [18]. Specifically, we found that every additional standard deviation of village-level community mobilization was associated with a 12% lower HIV incidence among AGYW enrolled in the HPTN 068 cohort in rural South Africa [18].

Little is known about the mechanisms linking CM to HIV incidence among young female residents. Pro-social community engagement may be one mechanism. The CM process is theorized to facilitate community participation by bringing communities together in solidarity to collectively work to achieve a shared goal [19]. As described previously, pro-social engagement during adolescence, such as participating in school or sports groups, has also been associated with reduced HIV risk behaviors [13]. School attendance may be another mechanism linking CM to reduced HIV incidence. CM domains such as social control have been associated with increased educational attainment [20], and school attendance has been inversely associated with HIV incidence among AGYW in South Africa [21]. Finally, a third mechanism may be AGYW’s hope for the future. The CM process may engender hope, as communities come together to address shared concerns and effect change. Hope for the future has also been inversely associated with HIV risk behaviors among AGYW in South Africa [22]. We examined these three hypothesized mediators of the relationship between CM and HIV incidence among AGYW in the HPTN 068 cohort in rural South Africa.

## Methods

### Setting and Procedures

We conducted a secondary data analysis using data from three data sources collected during our research with AGYW and their communities in the province of Mpumalanga, South Africa. The datasets include: (1) a longitudinal cohort of AGYW participating in the HPTN 068 trial, including survey and sero-prevalence data from the AGYW, and parenting and economic data reported by the heads of household; (2) two cross-sectional representative community surveys with surveys conducted in villages where the HPTN 068 cohort resided—the first survey occurred in 2012 and included 22 villages and the second was conducted in 2014 and included 26 villages; and (3) census data from the Agincourt HDSS site, where the HPTN 068 study and community surveys took place.

HPTN 068 was a phase III, randomized controlled trial of cash transfers conditional on school attendance among AGYW in the Bushbuckridge sub-district of the Mpumalanga province. The study area is within the Agincourt Health and Socio-Demographic Surveillance System (HDSS) study area, where the Medical Research Council and University of the Witwatersrand Rural Public Health and Health Transitions Research Unit conduct an annual census [23]. AGYW ages 13–20 enrolled in grades 8–11 and living in the research area were eligible to participate. All participants completed interviews and HIV testing at baseline (2011–2012), up to three annual follow-up visits (2012–2015) during the trial and an additional post-trial visit (2015–2017). Details of the HPTN 068 trial have been previously published [24, 25].

A cluster-randomized trial to test the effect of a CM intervention on harmful gender norms and HIV risk behaviors was implemented during the HPTN 068 trial in 22 villages (11 randomly selected to receive the intervention) in the Agincourt HDSS study area [26]. The CM intervention aimed to promote gender equitable norms and raise consciousness around the intersections of HIV and gender to reduce gender-based violence and improve HIV prevention behaviors and testing uptake. Intervention activities included 2-day intensive workshops led by trained community mobilizers; a range of community outreach activities; establishing and training volunteer cadres called Community Action Teams in each community; and engaging community leadership [26]. Intervention workshops were open to men and women (aged 18–35) in each intervention community and addressed seven content areas: gender, power, and health; gender and violence; alcohol; gender, HIV and AIDS; healthy relationships; human rights; and taking action for change. Mobilizers and Community Action Team members also led community outreach activities including door-to-door home visits, street soccer and soccer tournaments, mural design and discussions, and health talks. Formal leadership in each intervention community were engaged to discuss intervention themes and collaborate on activities applicable to the local context of each community. More details on the intervention content and design have been published elsewhere [26]. Two cross-sectional surveys were conducted to evaluate the 2-year CM intervention in 2012 (n = 1181), prior to the intervention, and in 2014 (n = 1403), after the intervention [26]. Adults aged 18–35 years were randomly sampled from the census population to participate in the surveys. The CM survey and sampling procedures have been described in detail elsewhere [26].

### Measures

**Fig. 1:**
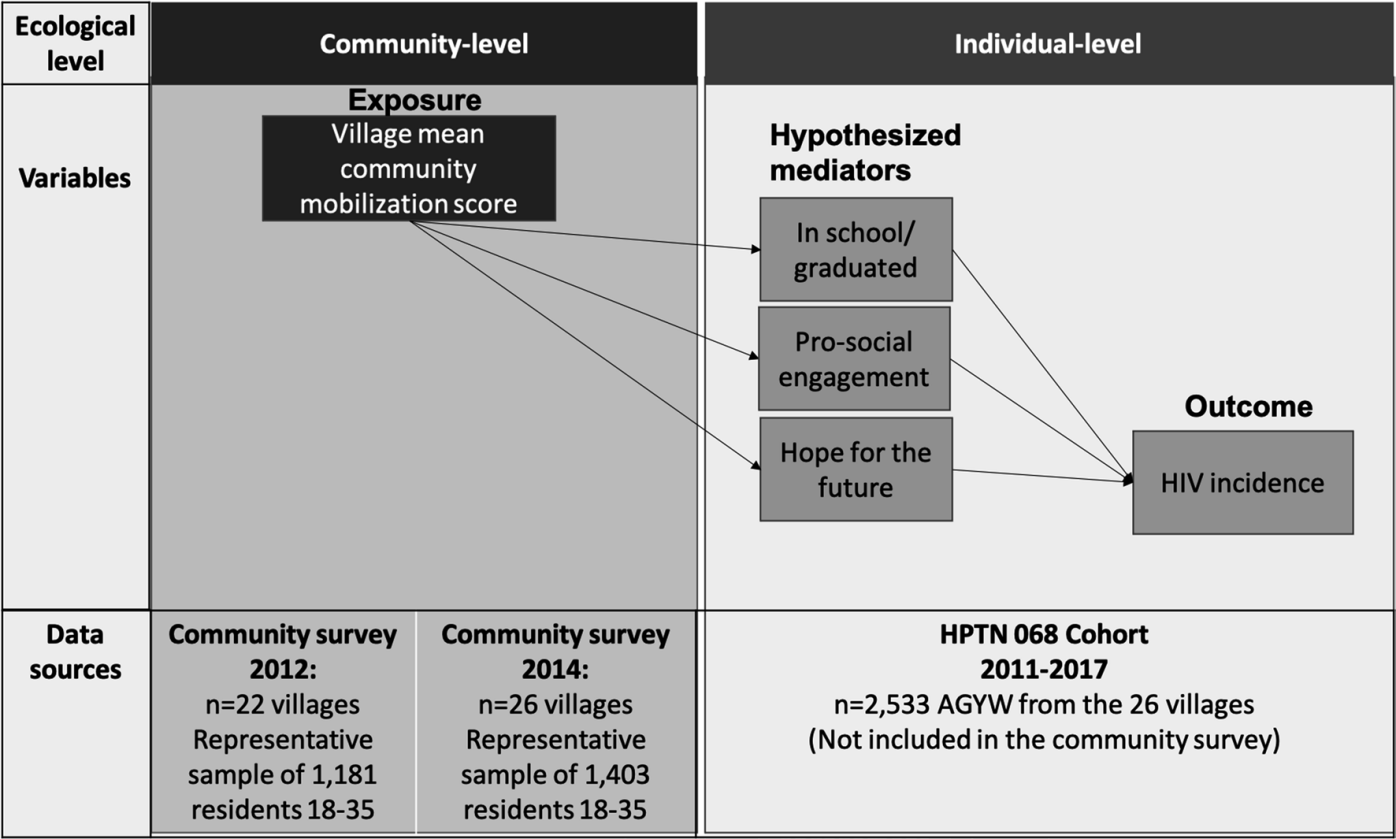
Study schematic of the exposure, mediators and outcome and the contributing data sources and timelines in Agincourt, South Africa

The variables of interest and their data sources are depicted in Fig. 1. The outcome was HIV status, which was assessed for each HPTN 068 cohort member at every study visit and was determined by parallel HIV rapid tests [24, 25]. The exposure, village CM, was measured at the community-level in both community surveys in 2012 and 2014. The CM measure comprised the seven domains described above. We aggregated individual responses into mean CM scores for each village, with higher scores indicating more mobilization. The development and validation of the CM measure has been described in detail [16].

The hypothesized mediators were AGYW pro-social engagement, hope for the future and school attendance measured for each HPTN 068 cohort member. Pro-social engagement was measured by a seven-item index that assessed whether (yes/no) participants belonged to seven different groups (e.g. sports team, student group etc.). Responses were summed to create a continuous score ranging from 0 to 7. Hope for the future was measured by a 13-item scale developed for AGYW in the HPTN 068 cohort (Cronbach’s α = 0.95) [22]. Example hope items include “I trust that I will be able to do everything that I want to do in my future” and “I believe the things I am doing now are preparing me for what I want in the future.” Participants rated how often they agreed with the statements in the items using a 4-point Likert scale (1 = rarely or none of the time; 4 = all of the time). Responses were summed to create a continuous score ranging from 1 to 52. The hope scale was log transformed to satisfy assumptions of normality. School attendance was operationalized as a dichotomous indicator of currently in school or graduated high school vs. not attending school or dropped out.

### Analysis Plan

Data from HPTN 068, the two community surveys, and the Agincourt HDSS census were merged so each HPTN visit was linked to the most recent preceding village data to ensure community (exposure) data preceded HIV outcome data, thereby preserving temporality. We restricted the dataset to participants who were HIV-negative at entry (n = 78 with prevalent HIV infection at baseline were excluded), and to those who reside in the villages included in the community survey (n = 159 participants were excluded due to no community survey data). Finally, we excluded four participants who became HIV infected prior to having community survey data to ensure temporal ordering (n = 4).

We previously estimated the total effect of village CM on HIV incidence, demonstrating a 12% lower HIV incidence with every standard deviation increase in village mobilization score [18]. To decompose the effect of village-level CM on HIV incidence directly versus through the hypothesized mediators, we examined the indirect effects of CM on HIV incidence using M*plus* 8.5, adjusting for relevant covariates and clustering of participants within villages. The indirect effect is the association of CM with HIV incidence through the mediator(s); a significant indirect effect indicates the presence of mediation. Because indirect effects are asymmetrically distributed, we then bootstrapped the 95% confidence intervals (CI) to obtain bias corrected 95% CIs [27]. Statistical significance of indirect effects was determined by whether the 95% CI included or excluded zero. Since the value for an indirect effect is zero under the null hypothesis, if the 95% CI excluded zero, the indirect effect was identified as statistically significant at *p* < 0.05.

Institutional Review Board approval for HPTN 068, the community surveys, and for merging the data sources for analysis was obtained from the University of North Carolina at Chapel Hill and the University of the Witwatersrand Human Research Ethics Committee. The University of California, San Francisco approved the community surveys and protocols for merging and analyzing de-identified data. All studies were conducted in accordance with the principles outlined in the Declaration of Helsinki.

## Results

The analysis included 2,292 AGYW from 26 communities. At enrollment, participants had a mean age of 15.5 years and 100% were in school (Table 1). By the end of follow-up, 88% had either graduated from high school or were still in school, and there were 194 incident infections. Community demographics did not change substantively over time.

**Table 1:** Baseline characteristics of HIV-negative adolescent girls and young women enrolled in HPTN 068 (n = 2292) and their communities (n = 26)

Participant characteristics	Baseline (n = 2292) n(%)	By the end of follow-up (n = 2225) n(%)
Mean age at entry into 068, (SD)	15.5 (1.6)	–
In school or graduated	2229 (100)	1961 (88.1)
Mean pro-social engagement score (range 0–7), (SD)	2.0 (1.3)	1.5 (1.0)
Mean hope for the future, logged (SD)	3.41 (0.3)	2.9 (0.6)
Any sexual intercourse	613 (26.8)	1,502 (67.5)
Engage in transactional sex in past 12 months^a^	72 (3.1)	548 (36.4)
Condomless sex in last three months^a^	189 (8.3)	699 (46.5)
HIV status		
HIV negative	2292 (100)	2031 (91.3)
HIV positive	0	194 (8.7)
Physical IPV in past 12 months	255 (10.4)	950 (41.32)
Ever pregnant^a^	200 (8.8)	931 (61.9)
Mean number of household assets, (SD) (asked about 27 durable goods)	14.0 (0.1)	15.6 (0.1)

Community characteristics^b^	Unweighted mean (SD) 2012	Unweighted mean (SD) 2014
Mean years of education	6.08 (0.61)	6.79 (0.49)
% permanent residents	62.36 (4.23)	59.81 (3.81)
Mean SES asset score	0.09 (0.54)	0.09 (0.52)

Community mobilization^c^	Weighted mean (SD) 2012	Weighted mean (SD) 2014
Total community mobilization score	2.22 (0.12)	2.15 (0.11)

^a^Among sexually active participants

^b^Data from Agincourt Health and socio-Demographic Surveillance System census

^c^Data from community surveys

Estimates of the mediation parameters between CM and HIV incidence are presented in Table 2. Hope for the future was found to mediate the relationship between CM and HIV incidence (indirect effect: RR: 0.98, bias-corrected 95% CI 0.96, 0.99, *p* < 0.05). Pro-social engagement and school attendance did not demonstrate indirect effects on the relationship between CM and HIV incidence, only direct effects (Table 2).

**Table 2:** Estimated direct and indirect effects of hypothesized mediators of village mean community mobilization score on HIV incidence in the HPTN 068 Cohort, Mpumalanga, South Africa, 2011–2017 (n = 2292)

Mediators^a^	Direct Effect (Bias corrected 95% CI)	Indirect Effect (Bias corrected 95% CI)
Girls’ pro-social engagement^b^	0.84 (0.73, 0.98)*	0.99 (0.98, 1.01)
Girls’ hope for the future	0.86 (0.74, 1.00)	0.98 (0.96, 0.99)*
In school/graduated high school	0.84 (0.73 0.99)*	0.99 (0.97, 1.00)

^a^Estimates were adjusted for household assets, HPTN intervention arm, community mobilization arm, age at baseline and community characteristics- a collated measure of three community-level variables (mean years of education, mean socio-economic status asset score, and proportion of the community who are permanent residents)

^b^Also adjusted for in school/graduated high school

*p < 0.05

## Discussion

Our group previously demonstrated that more mobilized villages were protective against HIV incidence among resident AGYW in rural South Africa (Box 1). In this manuscript, we explored hypothesized pathways linking village CM and HIV incidence among this population to better understand the mechanisms through which CM operates to prevent HIV acquisition risk and provide insight into targeted HIV prevention interventions. To our knowledge, this is the first study to find that hope for the future mediates the relationship between CM and HIV incidence. However, pro-social engagement and school attendance did not demonstrate mediation.

**Box 1:** What we have learned from past research

• Our group developed and validated a measure of community mobilization for HIV prevention in Mpumalanga Province, South Africa [16, 17]. Community mobilization comprises seven domains: shared concerns, critical consciousness, organizational structures/networks, leadership, collective action, social cohesion and social control. • We demonstrated, through a cluster-randomized trial, that community mobilization can reduce negative gender norms among men and has the potential to create environments that support IPV prevention and reduce HIV risk behavior among young people (aged 18–35) in Mpumalanga Province, South Africa [37]. • We also documented that village-level community mobilization was associated with a 12% lower HIV incidence among adolescent girls and young women (AGYW) residing in the village [18].	

Hope has been theorized by other scholars to be an important mediator between the larger social environment and engagement in HIV risk behaviors [28]. Bernays et al. (2007) argue that hope is a “measurable manifestation of the ways that social and economic structures function as risk regulators for the individual” [28]. In other words, some environments may engender hope for the future, providing the opportunity for individuals to consider the long-term consequences of their behaviors, while other environments may stifle hope and constrain individuals’ behaviors in such a way that gives rise to harmful behaviors and negative health outcomes [28]. Important here is individuals’ internalization of the structural factors that determine health opportunity and inequality [28]. Bernays et al. argue that acknowledging the role of hope in shaping health behaviors can inform the development of HIV prevention interventions that seek to create environmental conditions that foster hope [28]. However, complex concepts such as hope can be difficult to operationalize and therefore demonstrate their effects in research. Our study provides some of the first evidence highlighting the way in which hope for the future might influence HIV outcomes. Specifically, our findings suggest that CM, which includes residents’ perception of their community as cohesive and proactive, is internalized in young people. As a result, fostering mobilization is one way HIV prevention efforts can create a social environment conducive to hope and improve HIV risk reduction among AGYW.

Interestingly, we did not find that pro-social engagement or school attendance mediated the relationship between CM and AGYW HIV incidence, despite a theoretical basis for these pathways. It is possible that with only 194 incident infections, and 26 communities, we may have lacked the power to detect significant mediation for these hypothesized mediators [29]. It is also possible that CM has pervasive, yet diffuse impacts on a community and ensuant behaviors. While our findings suggest that CM leads to reduced AGYW HIV incidence, at least in part, through a path of hope, CM may also work through multiple additional paths (either at the community-, family-, or individual-level) that we are not measuring. For example, CM could be a marker of general community wellness, which, like other measures of ‘togetherness’ and ‘connectedness,’ can work through multiple pathways to influence health outcomes [30]. Community integration or “connectedness” has been posited to impact health outcomes by imbuing individuals with several forms of social support and resources, enforcing shared norms about health behaviors, and by facilitating a sense of attachment or belonging to ones’ community [30].

Drawing on this idea, CM may also be impacting girls’ HIV incidence indirectly, not through markers measured among the girls themselves, but through community members overall. The analyses conducted in this manuscript used CM measured in a representative sample in each village and the mediators and HIV incidence among AGYW residing in those villages. It is possible that CM may shape men’s (girl’s partners) behaviors, and it is men’s behaviors that are also impacting AGYW incidence. South African men engage in high levels of HIV risk behaviors including concurrent partnerships, alcohol use and IPV perpetration [31, 32]. These behaviors are, in part, driven by inequitable gender norms which value male “toughness” and power over women [33, 34]. Men who endorse such norms are more likely to engage in HIV risk behaviors and perpetrate IPV, which can increase their female partners’ HIV acquisition risk [31, 35, 36]. The CM intervention conducted by our team was designed to address inequitable gender norms [26] and significantly increased men’s endorsement of equitable gender norms in the intervention arm [37]. Thus, the protective effect of community mobilization on AGYW HIV incidence may reflect the impact of the CM intervention on men’s behaviors. Indeed, AGYW enrolled in the CM intervention reported reduced rates of IPV at follow-up compared to AGYW in the control [38]. Our past research has also demonstrated that community collective efficacy, a component of community mobilization, is associated with reduced incidence of IPV among AGYW in this setting [39].

## Conclusion

Our findings suggest that hope for the future is an important mediator of the impact of CM on AGYW HIV incidence. HIV prevention interventions that adopt a CM approach may alter the social environment in such a way that engenders AGYW hope for the future and enables them to engage in more HIV prevention behaviors. Future HIV prevention efforts targeted to AGYW in sub-Saharan Africa can benefit by adopting a CM approach to facilitate AGYW hope for the future. There is also a need to conduct additional research to further explore the remaining pathways linking CM to HIV incidence among AGYW in this setting, including larger sample sizes to ensure adequate power to detect differences. This research should also critically consider whether CM is impacting HIV incidence among residents through multiple diffuse pathways that may be shaping attitudes, behaviors, and norms at the community-level, including whether men’s HIV-related behaviors lie on the path between CM and AGYW HIV incidence. Only by better understanding the pathways linking a mobilized community to AGYW HIV incidence can we then consider the impacts of interventions to optimize HIV risk reduction among this vulnerable population.

## Data Availability

The HPTN 068 data we analyzed in this paper contains sensitive health information, including HIV status and sexual behavior data, of young women in rural South Africa. In accordance with the protocol of this secondary data analysis reviewed and approved by the University of California, San Francisco Institutional Review Board, we cannot make the dataset publicly available to third party users. However, data access is managed by FHI360 and data requests can be made by contacting Erica Hamilton at EHamilton@fhi360.org.
